# Beyond Systemic Inflammation: The Complementary Role of Cellular Stress Markers in Predicting STEMI Outcome. Comment on Magureanu et al. Early Inflammatory Biomarkers, Ventricular Dysfunction and In-Hospital Mortality in Patients with ST-Elevation Myocardial Infarction Undergoing Primary Percutaneous Coronary Intervention. *Diagnostics* 2026, *16*, 1978

**DOI:** 10.3390/diagnostics16172873

**Published:** 2026-09-07

**Authors:** Fikret Keles

**Affiliations:** Department of Cardiology, Kırşehir Training and Research Hospital, Kırşehir 40100, Türkiye; keles.fikret@gmail.com

I read with interest the recently published article by Magureanu et al. titled “Early Inflammatory Biomarkers, Ventricular Dysfunction and In-Hospital Mortality in Patients with ST-Elevation Myocardial Infarction Undergoing Primary Percutaneous Coronary Intervention” in your esteemed journal [1]. The investigators demonstrated the association between early systemic inflammatory indices (such as NLR, PLR, and CRP) and adverse short-term outcomes, including left ventricular dysfunction and in-hospital mortality, in patients with ST-segment elevation myocardial infarction (STEMI). Their comprehensive approach underscores the pivotal role of immune-inflammatory pathways in the acute phase of myocardial ischemia.

Current clinical guidelines primarily rely on high-sensitivity cardiac troponins (hs-cTn) for the diagnosis and risk stratification of acute myocardial infarction. However, while troponins are excellent indicators of the extent of myocardial necrosis, they have distinct limitations. They often fall short in capturing the full pathophysiological complexity of STEMI, particularly the intense systemic inflammatory and profound cellular stress responses that drive early hemodynamic complications.

Building upon their valuable findings, I believe it is crucial to expand the risk stratification paradigm to include markers of profound cellular stress, which act parallel to, and often precede, systemic inflammatory cascades. The extensive myocardial necrosis and severe endothelial perturbation inherent to STEMI are characterized not only by inflammatory cell infiltration but also by the acute release of intracellular chaperones into the systemic circulation.

In this context, the prognostic utility of Heat Shock Protein 60 (HSP60) warrants special attention. In my previous prospective cohort study involving 221 patients presenting with acute STEMI, I demonstrated that elevated admission levels of HSP60 (>7.325 ng/mL) at admission were strongly and independently associated with the development of in-hospital cardiogenic shock and short-term mortality, yielding a moderate predictive accuracy (AUC: 0.70, sensitivity: 67.7%) [2]. This aligns with the broader literature indicating that robust indices like the systemic immune-inflammation index (SII) and intracellular chaperones like HSP60 are critical drivers of adverse cardiovascular events and early deterioration [3,4].

While the authors highlight that inflammatory biomarkers primarily reflect the severity of myocardial injury, my findings indicate that HSP60 serves as a direct barometer of severe cellular distress and impending hemodynamic collapse. The significant association found between admission HSP60 values and the in-hospital development of heart failure or cardiogenic shock suggests that evaluating cellular stress markers alongside traditional inflammatory indices could further refine the early identification of the highest-risk STEMI phenotypes.

Therefore, I propose that future predictive models and clinical algorithms incorporate a dual-axis approach—integrating both systemic inflammatory ratios (as demonstrated in the authors’ study) and specific cellular stress markers like HSP60. Such an integrated multi-marker strategy could optimize early decision-making and overcome the limitations of relying solely on conventional biomarkers.

I congratulate the authors on their significant contribution to the field and look forward to future research exploring these complementary pathophysiological pathways.

## Data Availability

No new data were created or analyzed in this study. Data sharing is not applicable to this article.

## References

[1] Magureanu D.C., Hiceag M.L., Rus C.B., Ghitea T.C., Cinezan C. (2026). Early Inflammatory Biomarkers, Ventricular Dysfunction and In-Hospital Mortality in Patients with ST-Elevation Myocardial Infarction Undergoing Primary Percutaneous Coronary Intervention. Diagnostics.

[2] Karataş R., Çelik M., Yılmaz A., Keles F., Aygül N., Vatansev H., Sökmen E., Erseçgin A. (2020). The value of heat shock protein (HSP) 60 on in-hospital and short-term prognosis in patients with acute ST segment elevation myocardial infarction. J. Surg. Med..

[3] Öcal L., Keskin M., Cerşit S., Eren H., Çakmak E.Ö., Karagöz A., Çakir H., Gürsoy M.O., Doğan S., Zhalilov M. (2022). Systemic immune-inflammation index predicts in-hospital and long-term outcomes in patients with st-segment elevation myocardial infarction. Coron. Artery Dis..

[4] Bonanad C., Nunez J., Sanchis J., Bodi V., Chaustre F., Chillet M., Minana G., Forteza M.J., Palau P., Nunez E. (2013). Serum heat shock protein 60 in acute heart failure: A new biomarker. Congest. Heart Fail..

